# Clinical evidence for efficacy of pembrolizumab in MSI-H and TMB-H advanced solid tumor: results from three cancer centers in China

**DOI:** 10.1007/s00262-024-03660-2

**Published:** 2024-03-07

**Authors:** Huan Yan, Lianxi Song, Yizhi Li, Qinqin Xu, Wenhuan Guo, Shaoding Lin, Wenjuan Jiang, Zhan Wang, Li Deng, Zhe Huang, Haoyue Qin, Xing Zhang, Fan Tong, Ruiguang Zhang, Zhaoyi Liu, Lin Zhang, Juan Yu, Xiaorong Dong, Qian Gong, Jun Deng, Xue Chen, Jing Wang, Gao Zhang, Nong Yang, Yongchang Zhang, Liang Zeng

**Affiliations:** 1 Department of Medical Oncology, Lung Cancer and Gastrointestinal Unit, Hunan Cancer Hospital/The Affiliated Cancer Hospital of Xiangya School of Medicine, Central South University, Changsha, 410013 China; 2 Graduate Collaborative Training Base of Hunan Cancer Hospital, Hengyang Medical School, University of South China, Hengyang, 421001 Hunan China; 3 Department of Medical Oncology, Yiyang Central Hospital, Yiyang, 413000 China; 4 Department of Medical Oncology, Qinghai Provincial People’s Hospital, Xining, 810000 China; 5 Department of Pathology, Ninth People’s Hospital, Shanghai Jiao Tong University School of Medicine, Shanghai, 20025 China; 6 Department of Medical Oncology, The First Affiliated Hospital of Hunan University of Medicine, Huaihua, 418000 China; 7 Cancer Center, Union Hospital, Tongji Medical College, Huazhong University of Science and Technology, Wuhan, 430022 China; 8 Department of Medical Oncology, The Affiliated Cancer Hospital of Xiangya School of Medicine, Central South University/Hunan Cancer Hospital, Changsha, 410008 Hunan China; 9 Department of Radiotherapy, The Affiliated Cancer Hospital of Xiangya School of Medicine, Central South University/Hunan Cancer Hospital, Changsha, 410008 Hunan China; 10 Department of Medical Oncology, Zhangjiajie People’s Hospital, Zhangjiajie, 410008 Hunan China; 11 Department of Good Clinical Trials, The Affiliated Cancer Hospital of Xiangya School of Medicine, Central South University/Hunan Cancer Hospital, Changsha, 410008 Hunan China; 12 Early Clinical Trails Center, The Affiliated Cancer Hospital of Xiangya School of Medicine, Central South University/Hunan Cancer Hospital, Changsha, 410008 Hunan China; 13 Faculty of Dentistry, The University of Hong Kong, 34 Hospital Road, Sai Ying Pun, 999077 Hong Kong; 14 Furong Laboratory, Changsha, 410000 Hunan China

**Keywords:** Pembrolizumab, Microsatellite instability, Tumor mutational burden, MSI-H, Immune checkpoint inhibitor, Solid tumor

## Abstract

**Background:**

Pembrolizumab has been indicated in the treatment of solid tumors with high frequency microsatellite instability (MSI-H) or high tumor mutational burden (TMB-H); however, real-world data on the effectiveness of pembrolizumab with or without chemotherapy in this molecular subset remain limited. Our retrospective study evaluated the clinical efficacy and safety of pembrolizumab in treating advanced solid tumors with either MSI-H or TMB-H.

**Methods:**

This retrospective study analyzed data from 116 patients with MSI-H or TMB-H advanced solid cancers who received pembrolizumab with or without chemotherapy regardless of treatment setting. We analyzed objective response rate (ORR) and progression-free survival (PFS).

**Results:**

The top three cancer types were colorectal (48.6% MSI-H, 6.5% TMB-H), lung (15.4% MSI-H, 84.4% TMB-H), and gastric (15.4% MSI-H, 5.1% TMB-H). The ORR with pembrolizumab was 52.6%, including complete response (CR) observed in 8.6% (n = 10) of cases and partial responses (PR) in 43.9% (n = 51). Of the 93 patients who received first-line pembrolizumab, 52 patients achieved objective response (10 CR, 42 PR), with a median PFS of 14.0 months (95% confidence intervals [CI] 6.6–21.4). Of the 23 who received subsequent-line pembrolizumab, the ORR was 39.1%, disease control rate was 91.3%, and median PFS was 5.7 months (95% CI 3.9–7.5). Treatment-related adverse events were observed in 32 patients (27.6%), with no reported treatment-related fatal adverse events.

**Conclusion:**

Our study provides real-world evidence on the clinical effectiveness of pembrolizumab with or without chemotherapy in the treatment of patients with MSI-H and TMB-H advanced solid cancers.

**Supplementary Information:**

The online version contains supplementary material available at 10.1007/s00262-024-03660-2.

## Introduction

The development of immune checkpoint inhibitors (ICIs) has revolutionized the treatment landscape for patients with advanced solid tumors [1]. In 2015, Le et al. reported their preliminary findings demonstrating the robust response of patients with microsatellite instability-high (MSI-H) to pembrolizumab, an inhibitor of the immune checkpoint component programmed cell death 1 protein (PD-1), after the failure of conventional therapy [2]. The remarkable efficacy observed in their study expedited the approval of pembrolizumab for the treatment of adult and pediatric patients with unresectable/metastatic solid tumors with high-frequency microsatellite instability (MSI-H) or mismatch repair deficient (dMMR) by the United States Food and Drug Administration (FDA) in 2017 [2–4]. MSI-H tumors are known to have a high frequency of length alterations within simple repetitive DNA sequences known as microsatellites. The loss of function of certain DNA mismatch repair genes that play a significant role in the DNA repair pathway, including *MLH1*, *PMS2*, *MSH2*, and *MSH6*, leads to deficient mismatch repair (dMMR), which can subsequently cause MSI-H [5].

Clinical data reveal that MSI-H is most frequently observed in colorectal cancer (CRC), endometrial cancer, and gastric adenocarcinomas [5]. MSI status can be assessed indirectly as the loss of expression of any of the four MMR-related subunit proteins using immunohistochemistry (IHC) or directly using polymerase chain reaction (PCR)-based amplification of five to seven microsatellite markers with highly unstable mononucleotide repeat loci [6–8]. In recent years, computational analyses of tumor next-generation sequencing (NGS) data have enabled accurate and robust estimation of MSI status, yielding a 95–100% concordance rate with PCR [9]. It has been estimated that approximately 15% of patients with CRC have MSI-H tumors and could benefit from anti-PD-1 therapy [10–15]. An initial study evaluated the efficacy of pembrolizumab administered at 10 mg/kg every 2 weeks in 41 patients with MSI-H tumors (either CRC or non-CRC), as well as microsatellite stable CRC. The reported objective response rates (ORRs) for MSI-H CRC and MSI-H non-CRC were 40% (4/10 patients) and 71% (4/7 patients), respectively, compared to 0% (0/18 patients) for microsatellite stable CRC [2].

Besides MSI-H and dMMR, a high tumor mutational burden (TMB-H) is also a validated biomarker for response to anti-PD-1 therapy [16]. TMB is estimated using somatic mutation data generated from whole exome sequencing or targeted DNA-based NGS of tumor cells and typically expressed as mutations per megabases (Mb) of the genome coverage of the gene panel used for sequencing. TMB-H tumors are believed to have more immunogenic neoantigens than TMB-low tumors. Tumor neoantigens are recognized by the host T cells, which is critical in immunotherapy response [16]. In addition to its tissue-agnostic indication in the treatment of MSI-H and dMMR tumors, pembrolizumab has also been FDA-approved for the treatment of solid tumors with TMB-H (≥ 10 mutations/Mb) based on the Foundation One companion diagnostic assay [16, 17]. Despite the approval of ≥ 10 mutations/Mb as a cutoff for TMB, the optimal threshold for TMB-H in predicting response to ICI therapy may vary by cancer type and the gene panel used [16]. Although the clinical benefit of patients with certain types of tumors with pembrolizumab has been well-established [12, 17], efficacy data regarding MSI-H or TMB-H lung and gynecological cancers are inconclusive and controversial. To complement this area, we conducted a retrospective study assessing the efficacy of anti-PD-1 inhibition in various solid tumors, including gastrointestinal, lung, and gynecological cancers harboring either MSI-H or TMB-H.

## Patients and methods

### Patients

This retrospective study screened clinical data of a total of 2,652 patients who sought treatment at five centers, including Hunan Cancer Hospital, Cancer Center, Union Hospital, Tongji Medical College, and Zhangjiajie People’s Hospital between January 2019 and September 2023. Study inclusion criteria were as follows: (1) 18 years of age or older, (2) had an Eastern Cooperative Oncology Group (ECOG) performance status of 0 or 1, (3) having solid tumors with either MSI-H or TMB-H, and (4) received pembrolizumab either as a monotherapy or combined with chemotherapy for a minimum of two months regardless of treatment setting.

### Molecular assays

Biopsy samples were obtained from the patients’ tumor tissues. Tissue sections were subsequently processed as formalin-fixed paraffin-embedded blocks and micro-sectioned to create pathological slides for IHC analysis for assessing the expression of the four MMR proteins. A minimum of 50 ng of DNA extracted from tissue biopsy collected from the patients was subjected to NGS analysis. This involved the utilization of commercially available panels designed to target at least 300 genes associated with cancer. The sequencing was performed on a Next-Seq 500 platform (Illumina, San Diego, CA) with paired-end reads, and the targeted sequencing depth was set at 1000× . These procedures for somatic variant calling and the assessment of TMB and MSI were carried out in accordance with optimized protocols provided by Burning Rock Biotech in Guangzhou, China [9, 18, 19]. A cutoff of 10 mutations/Mb was used to classify TMB-H across cancer types. PD-L1 expression was assessed by IHC of FFPE samples using 22C3 (n = 88), SP263 (n = 5), E1L3N (n = 4), and 28–8 (n = 1) as described previously [20]. PD-L1 expression was expressed as tumor proportion score (TPS) for all cancer types except CRC. PD-L1 TPS is measured as the total number of PD-L1 expressing tumor cells divided by the total number of all tumor cells. In CRC, PD-L1 expression was expressed as combined positive score (CPS), which counts the PD-L1 expression from tumor cells, lymphocytes and macrophages.

### Treatment regimen

Pembrolizumab was administered at 200 mg every 21 days. Combination therapy was administered with physician’s choice of chemotherapy. All patients underwent radiological assessments using either computed tomography scanning or magnetic resonance imaging before initiating treatment and every 4 weeks starting from the initiation of ICI therapy until the treatment is discontinued due to toxicity or confirmed disease progression. Treatment efficacy was assessed in terms of ORR, disease control rate (DCR), PFS, duration of response (DoR), and overall survival (OS). ORR was defined as the proportion of patients achieving a CR or PR. DCR was defined as the proportion of patients achieving CR, PR or stable disease. PFS was defined as the time from first dose of the treatment to the first documented disease progression or death, or last follow-up, whichever occurred first. DoR was only assessed among the patients who achieved response and defined as the time from receiving the treatment regimen until disease progression is confirmed. OS was defined as the time when first-line therapy was administered until death or last follow-up. The response assessments were done according to the Response Evaluation Criteria in Solid Tumors (RECIST) version 1.1 by investigator assessment. Adverse events were assessed and classified according to the Medical Dictionary for Regulatory Activities version 21. The data cutoff date was September 30, 2023.

### Statistical analysis

Continuous variables were summarized as means and standard deviations or medians with range. Categorical variables were summarized by presenting the frequencies with their corresponding percentages. Kaplan–Meier analysis was used to estimate the survival functions and log-rank test to determine the difference in survival outcomes between groups. Hazard ratios and their corresponding 95% confidence intervals (CI) were calculated using cox regression. *P* value < 0.05 was considered statistically significant. All statistical analyses were performed as two-sided tests using SPSS software (version 26.0) or GraphPad Prism (version 8.0).

## Result

### Patient characteristics

**Fig. 1:**
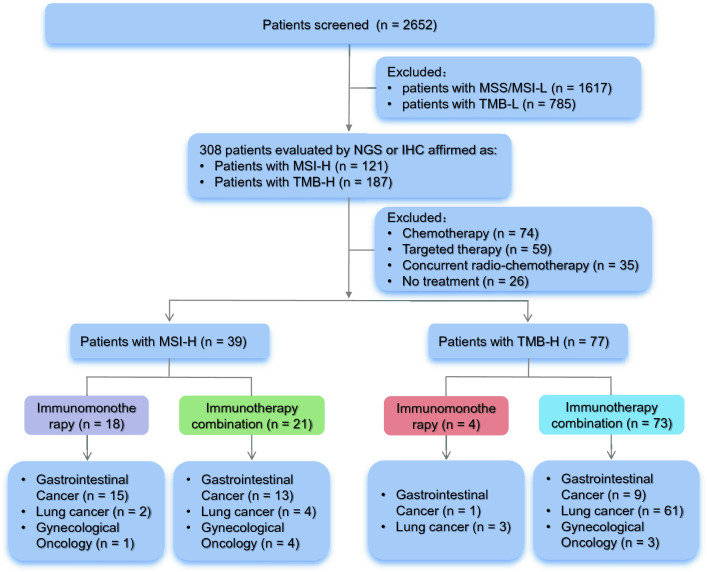
Flowchart of study

From a total of 2652 patient data screened, we identified 39 patients with MSI-H and 77 patients with TMB-H tumors who received pembrolizumab with or without chemotherapy (Fig. 1). Patients’ characteristics are presented in Table 1. It is worth noting that there were seven patients who were found to have MSI-H and TMB-H tumors. Of them, two patients had lung cancer, three patients had CRC, and a patient each had cervical cancer and appendiceal cancer. All these seven patients were included as MSI-H and were not included in the TMB-H analysis.

**Table 1:** Baseline demographics and disease characteristics

Characteristic	MSI-H (n = 39)	TMB-H (n = 77)
Age (year, median)	55	60
Range	21.0–78.0	26.0–81.0
Sex (n, %)		
Male	17 (43.6)	65 (84.4)
Female	22 (56.4)	12 (15.6)
Smoking history (n, %)		
Never	23 (59.0)	19 (24.7)
Former	16 (41.0)	58 (75.3)
ECOG performance status (n, %)		
0	12 (30.8)	22 (28.6)
1	27 (69.2)	55 (71.4)
Stage (n, %)		
III	10 (25.6)	17 (22.1)
IV	29 (74.4)	60 (77.9)
Cancer type (n, %)		
Gastrointestinal cancers		
Colorectal cancer	19 (48.6)	6 (7.8)
Gastric carcinoma	6 (15.4)	4 (5.2)
Appendiceal tumor	1 (2.6)	0 (0)
Pancreatic carcinoma	1 (2.6)	0 (0)
Thyroid carcinoma	1 (2.6)	0 (0)
Lung cancer	6 (15.4)	64 (83.1)
Gynecological cancers		
Endometrial cancer	3 (7.7)	0 (0)
Cervical cancer	2 (5.1)	2 (2.6)
Ovarian cancer	0 (0)	1 (1.3)
MSI/TMB assay methods (n, %)		
IHC	8 (20.5)	0 (0)
NGS	22 (56.4)	77 (100)
IHC+NGS	9 (23.1)	0 (0)
PD-L1 expression (n, %)		
< 1%	10 (25.6)	18 (23.4)
≥ 1%	7 (18.0)	31 (40.3)
Unknown	22 (56.4)	28 (36.3)
Location of metastasis (n, %)		
Liver metastasis	10 (25.6)	10 (13.0)
Bone metastasis	6 (15.4)	14 (18.2)
Lung metastasis	6 (15.4)	16 (20.8)
Treatment regimen administered (n, %)		
Pembrolizumab plus chemotherapy	21 (53.8)	73 (94.8)
Pembrolizumab monotherapy	18 (46.2)	4 (5.2)

The median age of patients with MSI-H tumors was 55 years (21.0–78.0 years); 56.4% (22/39) were females. According to cancer type, 71.8% (28/39) had gastrointestinal cancer, 15.4% (6/39) had lung cancer, and 12.8% (5/39) had gynecological cancer. Among the 17 patients with PD-L1 expression data, seven patients had positive PD-L1 expression, while ten patients had negative PD-L1 expression. Of the 31 patients who received pembrolizumab-containing regimen as first-line treatment, 38.7% (12/31) received pembrolizumab monotherapy, and the remaining patients received pembrolizumab combined with chemotherapy.

Of the 77 patients with TMB-H tumors, 84.4% (n = 65) were males and the remaining 12 patients were females. The median age was 60 years (26–81 years). Based on cancer type, 13.0% (10/77) had gastrointestinal cancer, 83.1% (64/77) had lung cancer, and 3.9% (3/77) had gynecological cancer. Of the 81.8% (63/77) who received pembrolizumab-containing regimen as their first-line treatment, 98.4% (61/63) received pembrolizumab combined with chemotherapy.

### Efficacy

**Fig. 2:**
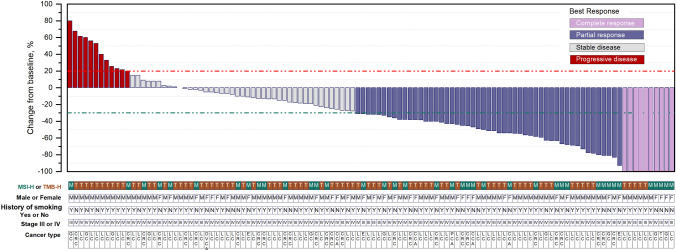
Waterfall plots summarizing the best change in tumor size (relative to baseline) for patients who received pembrolizumab with or without chemotherapy. The dotted line at 20% marks the threshold for evaluating progressive disease (PD), whereas the dotted line at − 30% marks the threshold for evaluating partial response (PR). Colors of the bars represent the best responses. Annotated below are patient characteristics, including MSI-H or TMB-H status, sex, smoking history, clinical stage, and cancer type

**Fig. 3:**
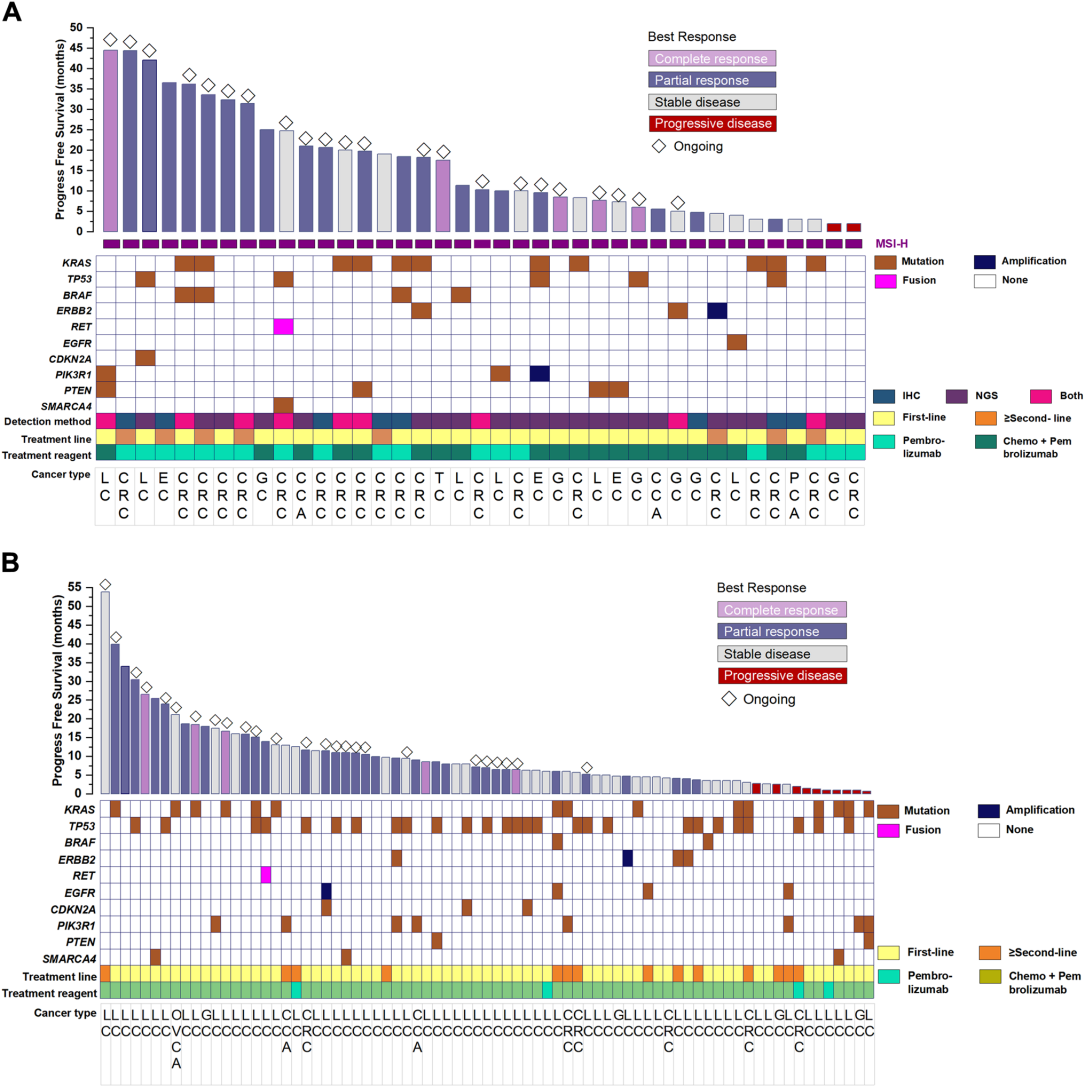
Bar plots showing the progression-free survival of patients with MSI-H (n = 39) (**A**) or TMB-H (n = 77) (**B**) who received pembrolizumab with or without chemotherapy. Each bar represents one patient. Colors of the bar represent the best response. Diamond on top of each bar denote ongoing treatment as of data cut-off date. Representative heatmap to indicate somatic mutation profile with colors indicating the mutation types such as indels, fusion and amplification). Also annotated below are patient characteristics, including treatment line, pembrolizumab with or without chemotherapy, MSI detection methods for MSI-H tumors, and cancer type

All 116 patients were included in the efficacy analysis and revealed an ORR of 52.6%, with 10 patients (8.6%) achieving CR and 51 patients (44.0%) achieving PR (Fig. 2). The responses were durable, with 38 patients (32.8%) maintaining response for more than 12 months (Fig. 3A, B). Of the patients who had at least one post-baseline assessment of tumor response, 81.0% (94/116) demonstrated a reduction in the size of their target lesions compared to baseline (Fig. 2). At the time of data cutoff, 37 patients (60.7%) had an ongoing response. Among the 10 patients who achieved CR, eight patients (80.0%) had an ongoing response (Fig. 3A, B).

From the somatic mutation profile in Fig. 3A, eleven patients with MSI-H harbored *KRAS* mutations. Of them, eight patients had CRC, one patient each with appendiceal cancer, endometrial cancer, and pancreatic cancer. PFS was comparable between patients with MSI-H tumors who harbored *KRAS* mutations and those who had wild-type *KRAS* (*p* > 0.05).

Among the 39 patients with MSI-H tumors, five patients had CR and 20 had PR, yielding an ORR of 64.1% (25/39). The median PFS was 36.5 months (95% confidence intervals [CI] 12.6–60.4 months), regardless of treatment regimen or treatment line (Supplementary Fig. 1A). Among the 25 patients who had objective response, the median DoR was 35.0 months (95% CI Not reached [NR]-NR) (Supplementary Fig. 1B). Among the 77 patients with TMB-H tumors, ORR was 46.7% (36/77), with five patients who had CR and 31 patients had PR. The median PFS was 8.5 months (95% confidence intervals [CI] 5.3–11.7 months), regardless of treatment regimen or treatment line (Supplementary Fig. 2A). Among the 36 patients who had objective response, the median DoR was 17.2 months (95% CI 4.8–29.6 months) (Supplementary Fig. 2B). As of the data cutoff date, 23 patients with MSI-H and 30 patients with TMB-H tumors were still receiving pembrolizumab therapy.

Among the 93 patients who received pembrolizumab with or without chemotherapy as first-line treatment, the median PFS was 14.0 months (95% CI 6.6–21.4 months) (Supplementary Fig. 3A) and the median OS was 22.0 months (95%CI: 16.0–28.0) (Supplementary Fig. 3B). PFS and OS outcomes were comparable regardless of treatment line (Supplementary Fig. 3A and B) or when receiving pembrolizumab monotherapy (Supplementary Fig. 4A). However, among the 94 patients who received pembrolizumab combined with chemotherapy, PFS was significantly longer for those who received the regimen as first-line therapy than as a later-line treatment (11.5 vs 4.5 months *P* = 0.002; Supplementary Fig. 4B). Additionally, PFS was also significantly longer among the 49 patients with PD-L1 positive tumors (PD-L1 TPS > 1%) than those with PD-L1-negative tumors (PD-L1 < 1%, 8.5 vs 4.5 months; *P* < 0.01; Supplementary Fig. 5).

We further assessed the efficacy of pembrolizumab according to cancer type. Patients with MSI-H non-CRC (n = 12) had a numerically longer PFS but was not statistically different from patients with MSI-H CRC (n = 27) (25.0 vs. 36.5 months, *P* = 0.639; Supplementary Fig. 6). The ORR among the MSI-H non-CRC was 83.3%. Among the patients with MSI-H non-CRC tumors, six patients had lung cancer, two patients had cervical cancer, and one patient had pancreatic cancer. Among them, two patients achieved CR, six patients achieved PR, and a patient had SD. Table 2 summarizes the details of the six patients with lung cancer with MSI-H. Notably, one patient with stage IV lung adenocarcinoma (patient number M24) had both MSI-H and TMB-H status, high PD-L1 expression (greater than 50%), and mutations in the *CDKN2A* and *TP53* genes. This patient achieved PR with pembrolizumab monotherapy as first-line regimen and still continues to benefit from the treatment even after 42.1 months (Table 2). Among the 64 patients diagnosed with TMB-H lung cancer, four patients demonstrated a PFS exceeding 30 months, while another three patients had a PFS surpassing 20 months, with an ORR of 50.8%. Table 3 summarizes the ORR and PFS according to cancer type.

**Table 2:** Detailed clinical and molecular characteristics of the six patients with MSI-H lung cancer

NO	Sex	Age	Smoking	Pathology diagnosis	Stage	MSI status detection	Driver Gene	*KRAS* mutation status	*TP53* mutation status	Other concomitant gene mutation	Metastasis	PD-L1 TPS (%)	TMB (mut/Mb)	Tissue sample assayed	Treatment setting	Treatment regimen	Best response	PFS (months)
M2	F	54	Never	LUAD	IV	NGS	*EGFR* mutation	WT	WT	WT	Lung, bone	70	Unknown	Primary tumor	First-line	Chemo+pembrolizumab	SD	4.0
M10	M	48	Former	LUAD	IV	NGS and IHC	WT	WT	WT	*MLH*, *PIK3R1*, *PRKDC*, *PTEN*, *TSC1*	Pleura	0	18.3	Primary tumor	First-line	Chemo+pembrolizumab	CR	44.5 +
M18	F	47	Never	LUAD	IV	NGS	*BRAF* mutation	WT	WT	WT	Lung, brain	0	4.18	Lymph node	First-line	Chemo+pembrolizumab	PR	11.3
M24	M	58	Never	LUAD	IV	NGS	WT	WT	Mutated	*CDKN2A*	Bone	50	26.12	Primary tumor	First-line	Pembrolizumab	PR	42.1 +
M25	M	68	Former	LUSC	IV	NGS	WT	WT	WT	*PIK3CA*	None	Unknown	Unknown	Primary tumor	First-line	Pembrolizumab	PR	10.0
M34	F	48	Never	LUSC	III	NGS	WT	WT	WT	*PTEN*	None	100	Unknown	Primary tumor	First-line	Chemo+pembrolizumab	CR	7.7 +

PFS values with plus  (+) symbols indicate that the patient was still receiving the treatment regimen as of data cutoff date

*Chemo* Chemotherapy; *CR* Complete response; *F* Female, *LUAD* Lung adenocarcinoma; *LUSC* Lung squamous cell carcinoma; *M* Male, *PD-L1 TPS* Programmed cell death-ligand 1 tumor proportion score; *PFS* Progression free survival; *PR* Partial response; *SD* Stable disease; *WT* Wild type

**Table 3:** Efficacy of pembrolizumab with or without chemotherapy based on cancer type

Cancer type	MSI-H (n = 39)		TMB-H (n = 77)	
	Pembrolizumab monotherapy (n = 18)	Pembrolizumab + chemotherapy (n = 21)	Pembrolizumab monotherapy (n = 4)	Pembrolizumab + chemotherapy (n = 73)
*Objective response rate*				
Gastrointestinal cancer	53.3% (8/15)	61.5% (8/13)	0% (0/1)	22.2% (2/9)
Lung cancer	100% (2/2)	75.0% (3/4)	0% (0/3)	54.1% (33/61)
Gynecological cancer	100% (1/1)	75.0% (3/4)	0% (0/0)	33.3% (1/3)
*Progression-free survival (months), median [95% confidence intervals]*				
Gastrointestinal cancer	Undefined	17.5 (5.86–29.14)	0	13.5 (NA-NA)
Lung cancer	26.05 (NA-NA)	27.9 (NA-NA)	0	15.9 (8.77–23.03)
Gynecological cancer	36.5 (NA-NA)	Undefined	0	11.7 (NA-NA)

### Safety

Of the 22 patients who received pembrolizumab monotherapy, eight experienced grade 1–2 immune-related adverse events (irAEs), including rash, elevated transaminase levels, pneumonitis, thyroid dysfunction, and myelosuppression. Additionally, three patients encountered grade 3 or above irAEs, including rash, elevated bilirubin, and colitis. Among the 94 patients who received pembrolizumab combined with chemotherapy, 36 patients experienced grade 1–2 irAEs. The most observed irAEs included elevated transaminase levels, abnormal thyroid function, and pneumonia. Grade 3 and above irAEs were observed in three patients, with mainly aminotransferase abnormalities and myositis. None of the patients required treatment discontinuation or experienced mortality related to adverse events (Supplementary Table S1).

## Discussion

This retrospective study assessed the efficacy and safety of pembrolizumab monotherapy or combined with chemotherapy in patients with advanced solid tumors exhibiting MSI-H or TMB-H. Our study included 116 patients with various solid cancer types, including cancer types such as colorectal and gastric cancers where the effectiveness of pembrolizumab had been established by various clinical trials. More importantly, our study also focused on the efficacy of pembrolizumab-containing regimen in less reported cancer types such as lung cancer and gynecological cancer. Our results indicate the encouraging prospect of using pembrolizumab in treating patients with advanced solid tumors characterized by MSI-H or TMB-H. The ORR was 52.6% and the DCR was 89.7%, revealing the effectiveness of pembrolizumab in treating these subsets of solid tumors. Importantly, when we observed comparable survival outcomes between patients with CRC and non-CRC with MSI-H. Given the diverse range of cancer types and our sample size, we acknowledge the need for larger datasets to corroborate these findings. We will also continue to follow up on the survival of this cohort. Nonetheless, pembrolizumab demonstrated favorable clinical outcomes in patients with MSI-H non-CRC, notably those with MSI-H lung cancer, which had an ORR of 83.3%.

Additionally, our study also focused on patients with TMB-H tumors. Currently, the optimal threshold for TMB-H is still inconclusive, with the threshold value varying across different cancer types and pathological classifications. We selected a cutoff value of 10 mutations/Mb to encompass five cancer types, including lung cancer, gastrointestinal, and gynecological cancer. Concurrently, we defined PD-L1 positivity as PD-L1TPS or CPS ≥ 1%. Our findings revealed that PD-L1-positive tumors had significantly longer mPFS than patients with negative PD-L1 expression. These results indicate that PD-L1 positive expression and TMB-H are associated with therapeutic response with pembrolizumab therapy.

Our findings support the efficacy data from the five pembrolizumab clinical trials (KEYNOTE-016, KEYNOTE-164, KEYNOTE-012, KEYNOTE-028 and KEYNOTE-158) [21]. Collectively, with a large real-world dataset of MSI-H and TMB-H cancers treated with pembrolizumab analyzed in this study, we further confirm the effectiveness of pembrolizumab with or without chemotherapy as first-line or subsequent-line therapy in patients with MSI-H and TMB-H solid tumors. These findings provide valuable insights for clinicians in making treatment decisions for patients with advanced solid tumors exhibiting MSI-H or TMB-H.

In terms of safety, only 32 out of all patients had experienced irAE, with only six cases who had grade 3 irAEs. Furthermore, none of the patients experienced treatment discontinuation due to adverse events. These results further support the favorable safety profile of pembrolizumab in patients with MSI-H and TMB-H advanced solid tumors.

Nonetheless, our study has certain limitations, including the relatively small sample size of patients with non-CRC. The existence of sampling bias might also confound our conclusion, as the patients who can access molecular testing and treatment regimens are included in this retrospective study, such as the increased use of NGS in lung cancer for detecting actionable somatic mutations has resulted in the inclusion of more patients with lung cancer in the TMB-H cohort, and the increased use of MSI and MMR assessments in CRC diagnosis has resulted in the inclusion of more patients with CRC in the MSI-H cohort. It is necessary to conduct studies with larger sample sizes and wider range of cancer types. There remains an urgent need to explore biomarkers to optimize treatment options.

In conclusion, our data provided real-world evidence of the effectiveness and safety of pembrolizumab in treating advanced solid tumors with MSI-H and TMB-H, providing valuable insights for clinicians to make treatment decisions in this subset of patients.

### Supplementary Information

Below is the link to the electronic supplementary material.

Supplementary file1 (PDF 268 KB)

## Data Availability

The datasets generated during and/or analyzed during the current study are available within the article and supplementary data files. The raw data are available from the corresponding author on reasonable request.

## Abbreviations

CR: Complete response
CRC: Colorectal cancer
dMMR: Mismatch repair deficient
ECOG: Eastern Cooperative Oncology Group
FDA: US Food and Drug administration
ICI: Immune checkpoint inhibitor
IHC: Immunohistochemistry
irAE: Immune-related adverse events
MSI-H: High-frequency microsatellite instability
NGS: Next-generation sequencing
ORR: Objective response rate
OS: Overall survival
PD-1: Programmed cell death 1 protein
PD-L1 TPS: Programmed death-ligand 1 tumor proportion score
PFS: Progression-free survival
PR: Partial response
TMB-H: High tumor mutational burden
