# Supplementary material for: Clinical evidence for efficacy of pembrolizumab in MSI-H and TMB-H advanced solid tumor: results from three cancer centers in China
Source: Cancer Immunol Immunother. 2024 Mar 7;73(4):74. doi: 10.1007/s00262-024-03660-2 (PMC10920474; doi:10.1007/s00262-024-03660-2)
Supplement: Supplementary file 1 — Supplementary file1 (PDF 268 KB) [file 262_2024_3660_MOESM1_ESM.pdf]

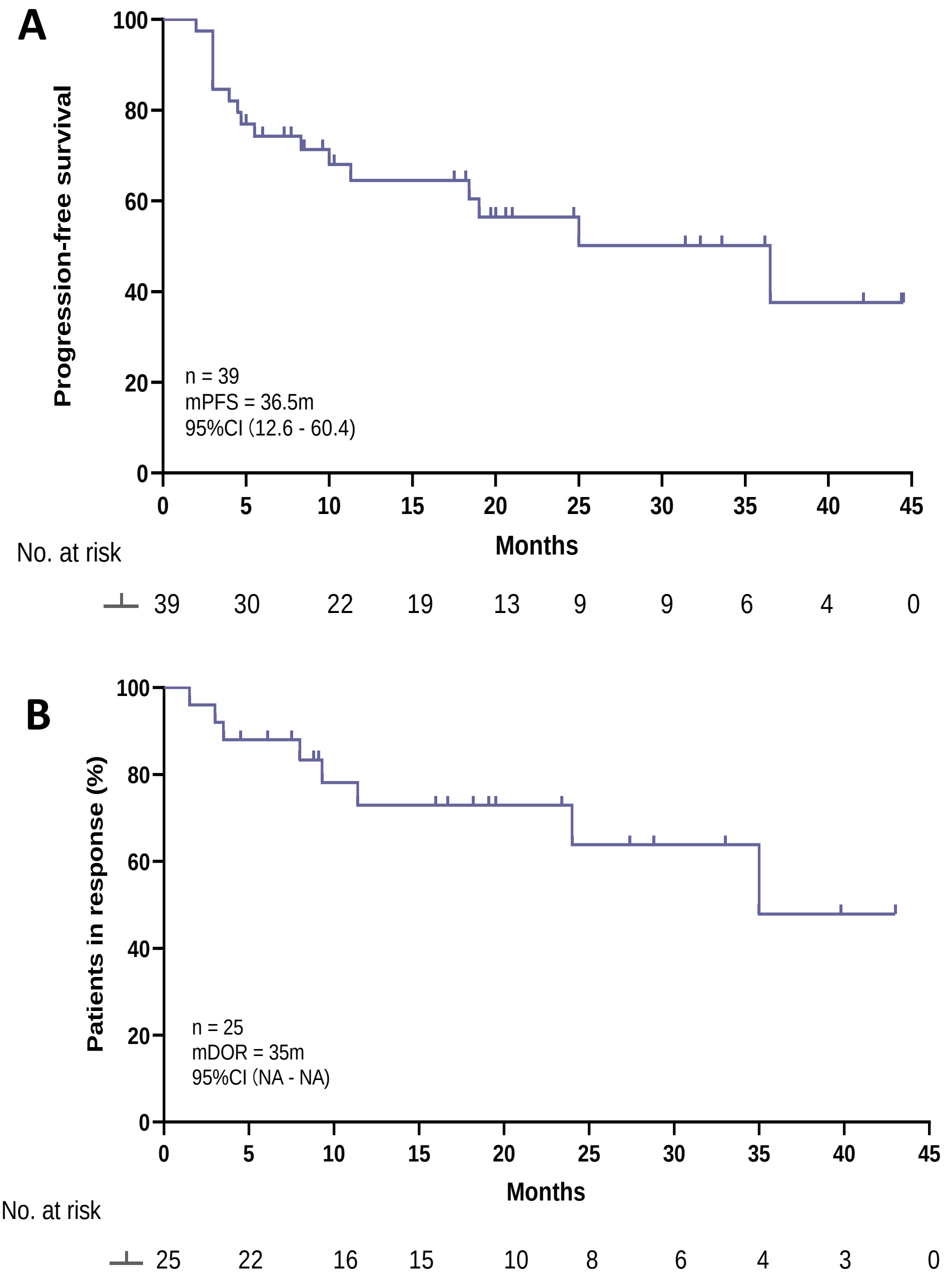

Figure S1. Kaplan-Meier curves plotting the progression-free survival (PFS) (A) and duration of response (DoR) (B) in patients with MSI-H tumors. Tick marks denote censored data. Risk table below indicates the number of events included per time point. The median PFS (mPFS) and median DoR (mDoR) and their corresponding 95% confidence intervals (CI) were indicated in the plots.

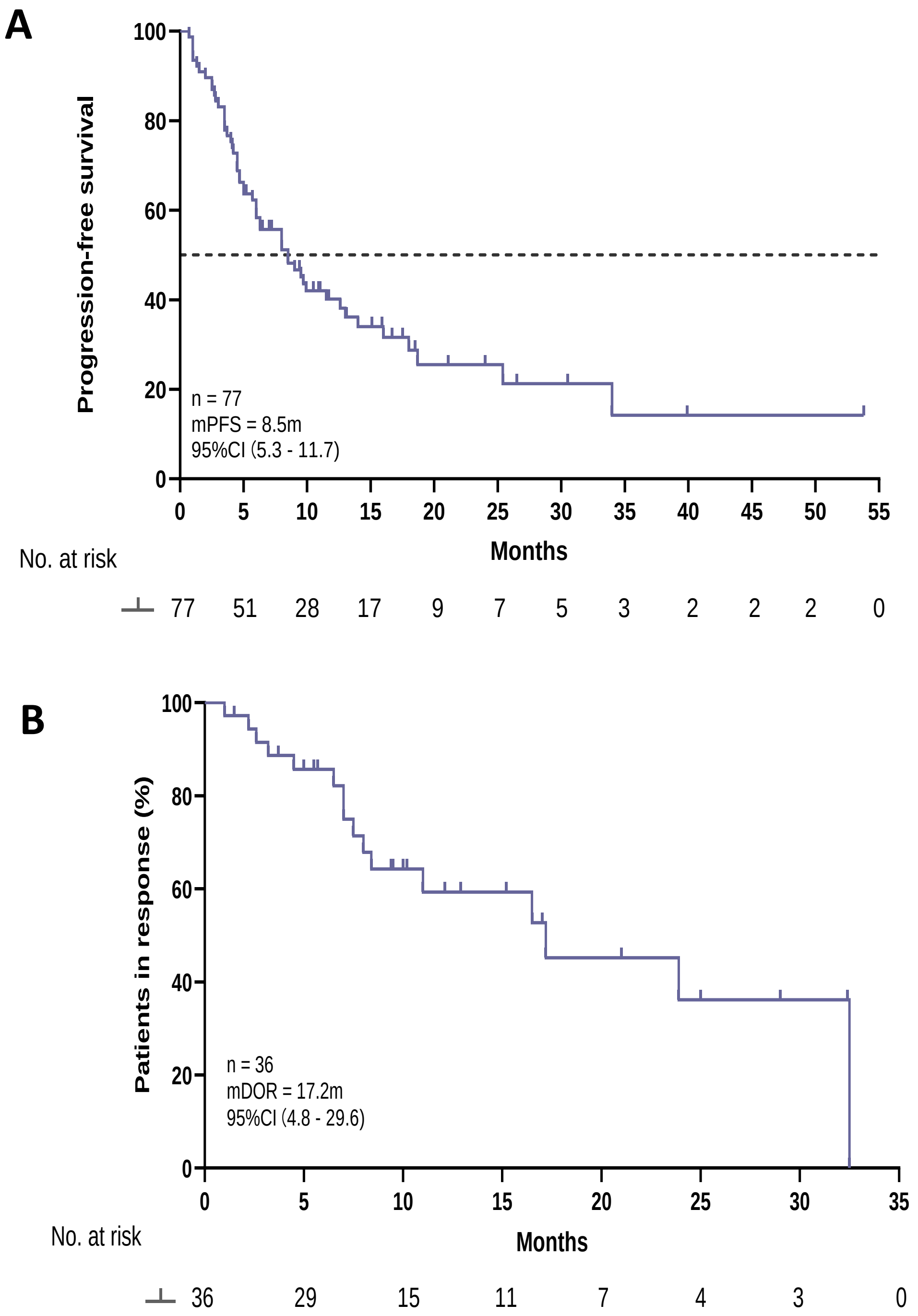

Figure S2. Kaplan-Meier curves plotting the progression-free survival (PFS) (A) and duration of response (DoR) (B) in patients with TMB-H tumors. Tick marks denote censored data. Risk table below indicates the number of events included per time point. The median PFS (mPFS) and median DoR (mDoR) and their corresponding 95% confidence intervals (CI) were indicated in the plots.

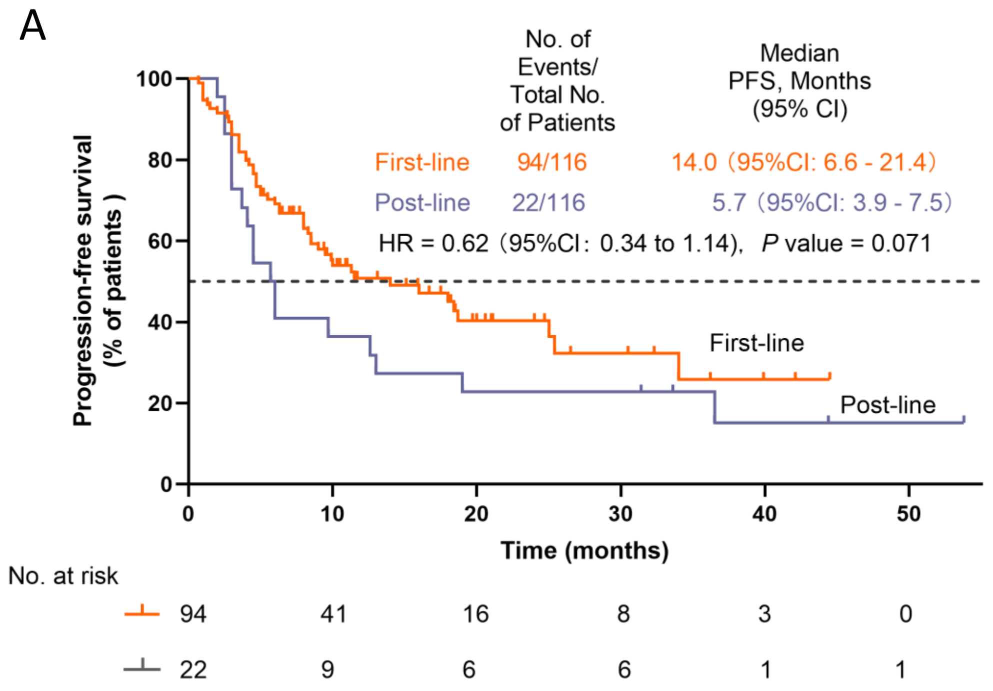

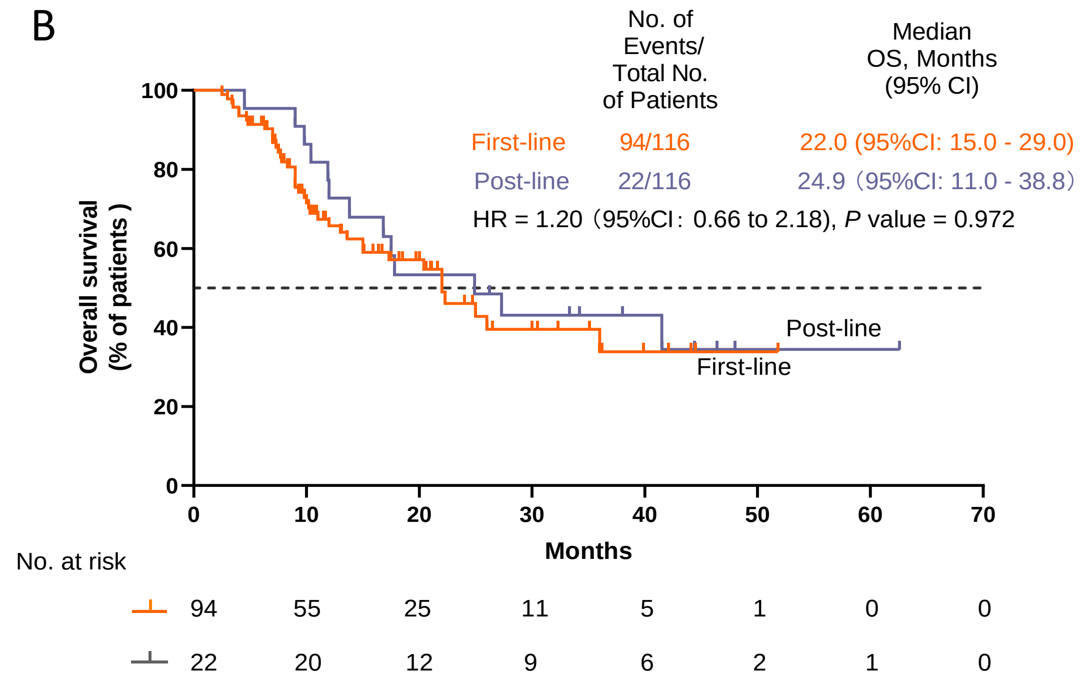

Figure S3. Kaplan-Meier curves comparing the progression-free survival (PFS) (A) and overall survival (OS) (B) of the patients who received first-line and post-line pembrolizumab-containing regimen. Tick marks denote censored data. Risk table below indicates the number of events included per time point. The median PFS and median OS, hazard ratios, and their corresponding 95% confidence intervals (CI) were indicated in the plots.

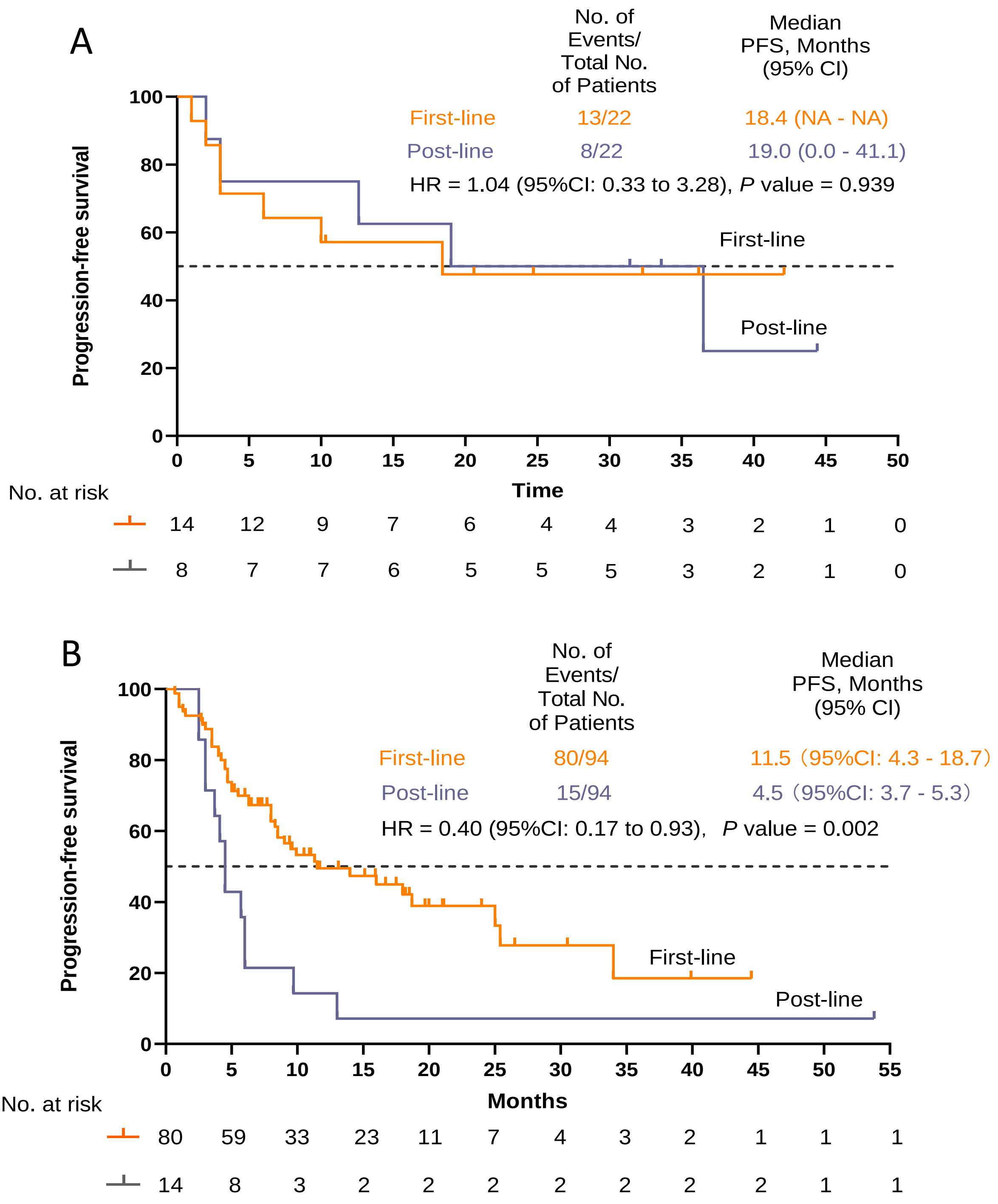

Figure S4. Kaplan-Meier curves comparing the progression-free survival (PFS) of the patients who received first-line and post-line pembrolizumab monotherapy (A) or first-line and post-line pembrolizumab combined with chemotherapy (B). Tick marks denote censored data. Risk table below indicates the number of events included per time point. The median PFS, hazard ratios, and their corresponding 95% confidence intervals (CI) were indicated in the plots.

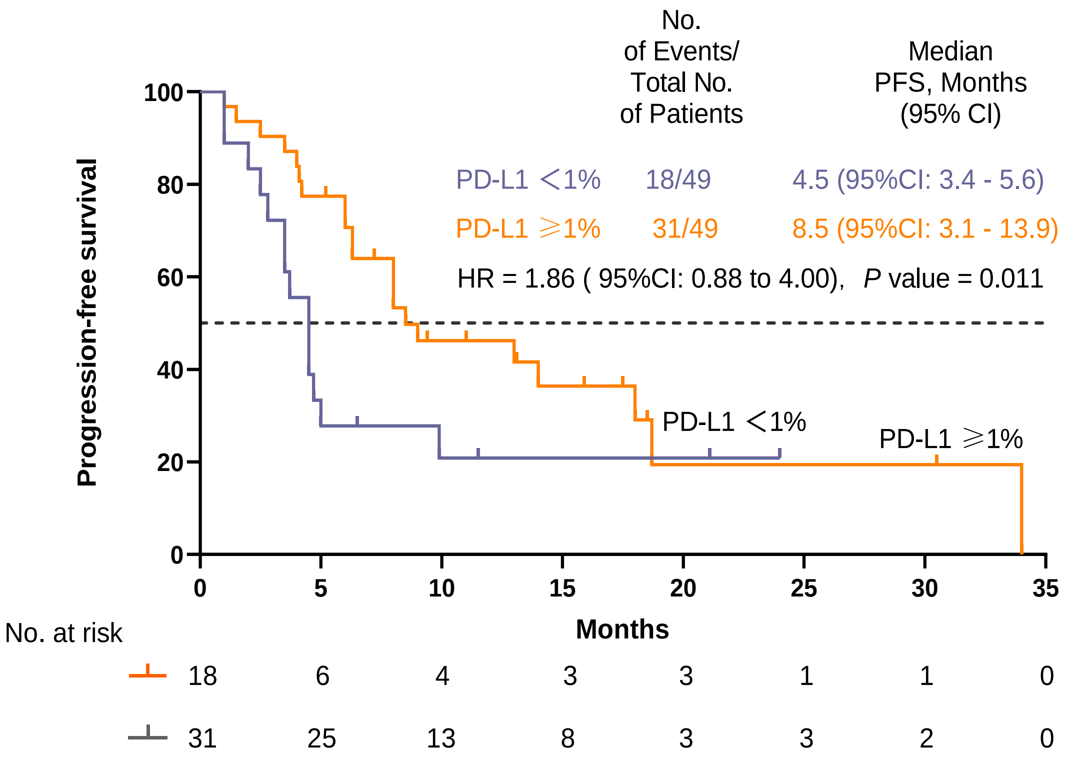

Figure S5. Kaplan-Meier curves comparing the progression-free survival (PFS) of the patients with TMB-H tumors stratified according PD-L1 expression as <1% and ≥1%. Tick marks denote censored data. Risk table below indicates the number of events included per time point. The median PFS, hazard ratios, and their corresponding 95% confidence intervals (CI) were indicated in the plot.

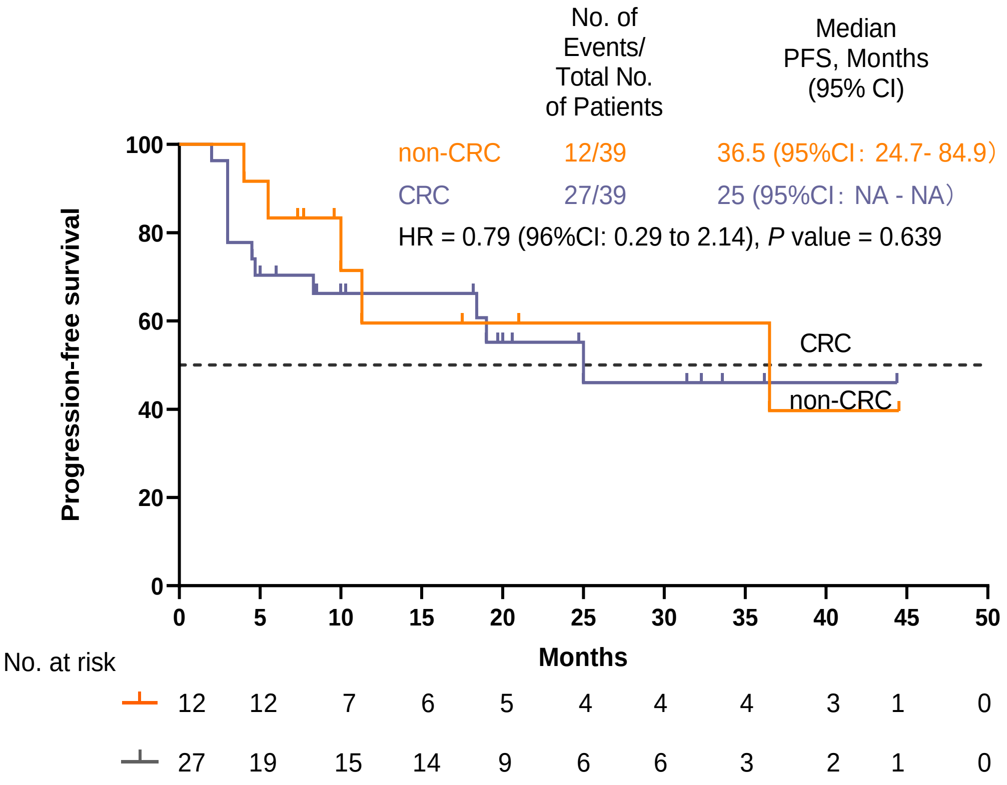

Figure S6. Kaplan-Meier curves comparing the progression-free survival (PFS) of the patients with MSI-H tumors stratified according to tumor type as colorectal cancer (CRC) or non-CRC. Tick marks denote censored data. Risk table below indicates the number of events included per time point. The median PFS, hazard ratios, and their corresponding 95% confidence intervals (CI) were indicated in the plot.

Table S1. Immune-related adverse events reported in patients with MSI-H and TMB-H who received pembrolizumab monotherapy or pembrolizumab combined with chemotherapy.

	**Pembrolizumab monotherapy (n = 22)**				**Pembrolizumab + chemotherapy (n = 94)**			
	**Grade 1-2**		**Grade ≥3**		**Grade 1-2**		**Grade ≥3**	
**Adverse events**	**No.**	**%**	**No.**	**%**	**No.**	**%**	**No.**	**%**
**Nonhematologic**								
Atrophy pancreas	0	0	0	0	1	1.1	0	0
Skin toxicity (rash)	1	4.5	1	4.5	1	1.1	0	0
ALT increased	3	13.6	0	0	13	13.8	1	1.1
Bilirubin increased	0	0	1	4.5	4	4.3	0	0
Pneumonia	1	4.5	0	0	5	5.3	0	0
Colitis	0	0	1	4.5	1	1.1	0	0
Thyroid dysfunction	1	4.5	0		6	6.4	0	0
Myocarditis	0	0	0	0	1	1.1	0	0
Myositis	0	0	0	0	1	1.1	2	2.1
**Hematologic**								
WBC decreased	1	4.5	0	0	2	2.1	0	0
Neutrophil decreased	1	4.5	0	0	0	0	0	0
Hemoglobinemia	0	0	0	0	1	1.1	0	0

Note: Adverse events are defined according to Medical Dictionary for Regulatory Activities (MedDRA) preferred terms.

Abbreviations: ALT, alanine aminotransferase; WBC, white blood cell.
